# Things Observed in New York by a Texas M. D.

**Published:** 1896-02

**Authors:** 


### Correspondence.

### Things Observed in New York by a Texas M. D.

December, 30, 1895.

*Editors Texas Medical Journal:*

Having just returned home, I find a reminder of one year's dues for the dear JOURNAL, which is herewith enclosed, and a hasty notice of things observed during my visit to the prominent hospitals of this country, by the handiwork of some of the great men—men great by achievements won, by character established, and by the spirit of philanthropy and good will as evidenced in their every day life. Six weeks of the six months from home was spent at the New York Polyclinical Hospital, the interesting events of which we will notice under the head of general surgery; beginning in the "Minor class" of work, the cure of "ingrown toe nails," which consists of, in the *extreme* radical method (removal of entire nail with its root) to that more feasible act of removal of the offending part of nail (*for present relief*) and the root of same (that *prevents recurrence* of the painful malady). Amputation of the toes, resection for bunions, "varus talipes" operations. Achileotomy and various tenotomies to correct deformities. Osteotomies to rectify abnormalities in the way of gneis valgum, etc. Fracture of patellæ approximated and retained by peculiar shaped adhesive plaster after the shape here given in cut. One section being applied above and another below the knee, retained in position by roller bandage, and the fragments drawn together by means of the buckle, which is so adjusted as to pass over the patellæ as in cut.

Resection at knee and hip joints: Amputation at hip joints by the bloodless method of Prof. John A. Wyeth, and as demonstrated by the author in this case, means as the name implies—bloodless *absolutely*, so far as arterial blood is concerned. In this case the operation was resorted to; to get rid of osteo-sarcoma of lower third of femur, the subject, a robust young man, could safely bear the loss of residual blood in the limb after it had been mantained iu elevated position five minutes. The after condition, so far has been uneventful recovery.

Castration to relieve enlarged prostate gland, is believed to be advantageous. The high operation being thought favorable of by many, was many times repeated.

Radical hernia operation—the Besseni, the MacEwens and combined methods.

Appendectomy—Oft repeated, not seldom by hernia followed.

Intestinal anastomoses by the Murphy-button, in case of fæcal fistulæ following operation to relieve suppurating appendicitis complicated by intestinal necrosis. Ovariectomy for very apparent reason, and in some instances the justification could only be understood by the *specialist.*

Multitudinous hysterectomies and not a few causes, occasionally what appeared to a student a *normal* uterus was the result.

The operation is one of great interest to your correspondent, inasmuch as it was successfully performed several times before having witnessed the operation at the hands of another.

The vaginal mood of the operation was performed under similar circumstances. Having witnessed the latter operation many times repeated by well known operators, I entertained adverse ideas in regard to the permissibility of the operation from the time of my effort, possibly on account of losing the patient, but I have only witnessed two satisfactory operations of the kind—one by Prof. Howard Kelly, the account of which I have not permission to submit.

The other by Prof. Goffe, an account of which is submitted somewhat in extenso as being one of the few, where this much lauded operation can be accorded the preference over the abdominal route.

There are a few specialists in this line that may and do produce very satisfactory results, and statistics following this procedure are uncertain; yet when considered by the general surgeon, who with temporary assistants and other disadvantages to be contended with, that of the few suitable vaginal canals, and the fewer advantageously positioned uteri and appendages to be removed, I believe the voice of a majority bear me out when I say celio-hysterectomy is by all odds more independent of individual conditions and more safely and satisfactorily performed.

Supra-pubic cystotomy is as satisfactory in results to the patient as its performance is simplicity to the operator.

Nephrectomy by the lumbar incision is most generally resorted to, size and position of the organ being governing factors, as I notice there is no hesitancy in entering the peritoneal cavity in case of large tumors of these organs.

The use of the Murphy button gives satisfactory results in cholecystenterostomy with many operators; however, there is quite an inclination to depend upon effecting intestinal anastomoses by Lembert mode of suturing.

Empyema is treated by resection of rib, though where a great quantity of fluid is present and the patient much exhausted, I have noticed that death followed the free, complete and instantaneous evacuating act, where not preceded by withdrawal of small quantities of fluid interruptedly.

Mention is here made of an interesting case of foreign body in the air passages. A man with suicidal intent severed the trachea and inflicting a deep ugly wound. Timely aid, and a achea tube prevented death. After-efforts failed to repair the damage done to the trachea and the tube was worn continuously to his admission into the hospital, the instrument was worn in two, the tube-end passing down into the bronchi.

Prof. Geo. Fowler located the tube in the right bronchi, through the medium of telephone probe; and by a bullet forceps being cautiously introduced, passing through the lumen of the tube, the jaws of forceps distended, the instrument was withdrawn incased by the tube. The position of the tube appeared to be just beneath the right nipple. The ingenuity here displayed by Prof. F., and the success attained was warmly applauded by all present.

In the various operations, witnessed on the superior extremity, the one that excited the most intense and universal interest was that of a young man affected by sarcoma of upper third of humerus. The chances being so unfavorable in efforts at a radical operation, the humanitarian surgeon proposed to inoculate the sanguinous growth with staphaloccus pyogonese aurem.

In effecting an opening in the growth rendering an avenue for ingress of the intended inoculation plan, the hemorrhage being so free that forcible packing of gauze into the large cavity of the growth caused by the breaking down of cancellated structure was resorted to, the hemorrhage being uncontrollable. Prof. Wyeth proceeded to ligate the subclavian, the supra-scapular and transversallis coli arteries by cocaine anesthesia, which he accomplished to the satisfaction of the patient, the operator and the class. Some days elapsed when the shoulder joint amputation by the bloodless method was done, as devised and practiced by Prof. Wyeth (previous to his performing the same operation upon the hip joint), he being the originator of the operation for both regions. The patient is now so markedly improved that he walked from the ward into the operating room (the ward for general surgery being adjacent to the amphitheatre) and exclaimed: "I am here yet, the only trouble is that 'blamed' nurse gave the egg-nog to that other man this morning."

The wound is granulating and a prospective chance seems to dawn.

The consideration that Prof. Wyeth extends to these poor sufferers can not fail to mark the greatness of the man to the most unobserving.

Even this meagre and imperfect synopsis, I find has grown too long, and I will stop short by saying that the faculty of teachers are as approachable and ever ready to accommodate, as compe-

*

tent and faithful, as can be found, yet I must particularize by saying "long live the philanthropist, the scientist, the country doctor's friend, the man of peace and good will to all—Professor John A. Wyeth."

SUBSCRIBER.

